# Supplementary material for: Non-Amontons frictional behaviors of grain boundaries at layered material interfaces
Source: Nat Commun. 2024 Nov 2;15:9487. doi: 10.1038/s41467-024-53581-y (PMC11531579; doi:10.1038/s41467-024-53581-y)
Supplement: Supplementary file 1 — Supplementary Information [file 41467_2024_53581_MOESM1_ESM.pdf]

Supplementary Information:

Non-Amontons Frictional Behaviors of Grain Boundaries at Layered Material Interfaces

Yiming Song,[†,1] Xiang Gao,[†,2] Rémy Pawlak,[1] Shuyu Huang,[1,3] Antoine Hinaut,[1] Thilo Glatzel,[1] Oded Hod,[*,2] Michael Urbakh,[*,2] Ernst Meyer[*,1]

[1] Department of Physics, University of Basel, Basel 4056, Switzerland.

[2] Department of Physical Chemistry, School of Chemistry, The Raymond and Beverly Sackler Faculty of Exact Sciences and The Sackler Center for Computational Molecular and Materials Science, Tel Aviv University, Tel Aviv 6997801, Israel.

[3] Key Laboratory for Design and Manufacture of Micro-Nano Biomedical Instruments, School of Mechanical Engineering, Southeast University, Nanjing 211189, China.

*Corresponding authors: Email: urbakh@tauex.tau.ac.il, odedhod@tauex.tau.ac.il, and ernst.meyer@unibas.ch

In this supplemental material, we provide additional details regarding the following aspects of the study reported in the main text:

1. Sample Preparation
2. Tip Preparation and Characterization
3. Determination of Grain Boundary Angle
4. Characterization of Additional Grain Boundaries
5. Energy Dissipation Route and Average Friction Calculation
6. Reproducibility and Additional Grain Boundary Friction Measurements
7. Molecular Dynamics Simulations
8. Two-State Model Parameter Fitting

Supplementary Note 1. Sample Preparation

The Pt(111) surface was cleaned by cycles of Ar^+ ion sputtering (energy of 1.2-1.8 keV under a preparation chamber pressure of 3×10^{-6} mbar for 10 min) and high temperature annealing using a home-made radio frequency heater. The surface was exposed to oxygen atmosphere (under a preparation chamber pressure of 5×10^{-8} mbar for 15 min) prior to the first annealing cycle to remove the carbide impurities. During the annealing procedure, the system was heated at a temperature of 1,400 K for a period of 20 min. Following this surface preparation step, monolayer polycrystalline graphene (PolyGr) was grown by means of high-temperature flash in ultrahigh vacuum (UHV). The sample was heated to a temperature of 1,073 K, and high purity ethylene was dosed onto the hot Pt(111) surface directly using a nozzle for 2 min under a preparation chamber pressure of 2×10^{-8} mbar. After that, the sample was kept at 1,073 K for an extra 20 min, followed by a slow cooling down process to room temperature at a rate of $1\ \mathrm{K}\cdot\mathrm{sec}^{-1}$. Supplementary Figure 1 presents an example of a non-contact atomic force microscope (NC-AFM) topography map of (a) an atomically clean Pt(111) surface prior to graphene growth, and (b) the same surface (but different location) after the graphene growth process.

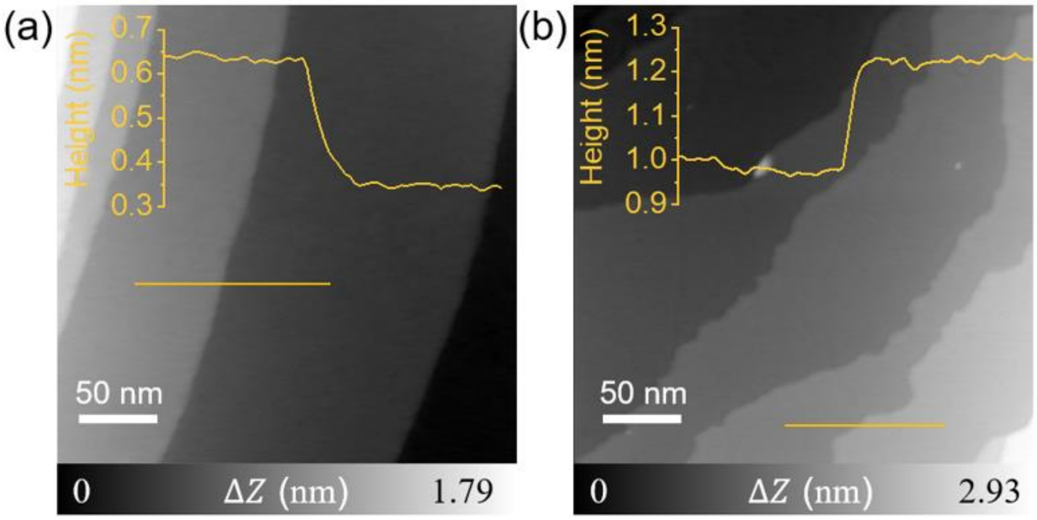

Supplementary Figure 1. Sample surface characterization. Topographic images of an atomically clean Pt(111) surface (a) prior to and (b) following graphene growth. Insets show the height profiles across a single atomic step edge (orange curves) measured by NC-AFM. Measurement parameters: $A_{1st} = 3$ nm and $\Delta f_{1st} = -14$ Hz in panel (a) and $A_{1st} = 3$ nm and $\Delta f_{1st} = -15$ Hz in panel (b).

Supplementary Note 2. Tip Preparation and Characterization

The AFM probe (PPP-CONT, Nanosensors) used for the friction measurements was annealed in UHV at a temperature of 473 K for several hours to remove residual contaminants from the tip surface prior to performing the friction force measurements. This was followed by Ar^+ ion sputtering of the tip for 2 min to remove the native silicon dioxide covering the tip. The normal and lateral forces acting on the probe were calibrated according to the procedures described in Ref. 1. Scanning electron microscopy (SEM) was used to determine the dimensions of the cantilever and tip radius (typically less than 7 nm), as shown in Supplementary Figure 2.

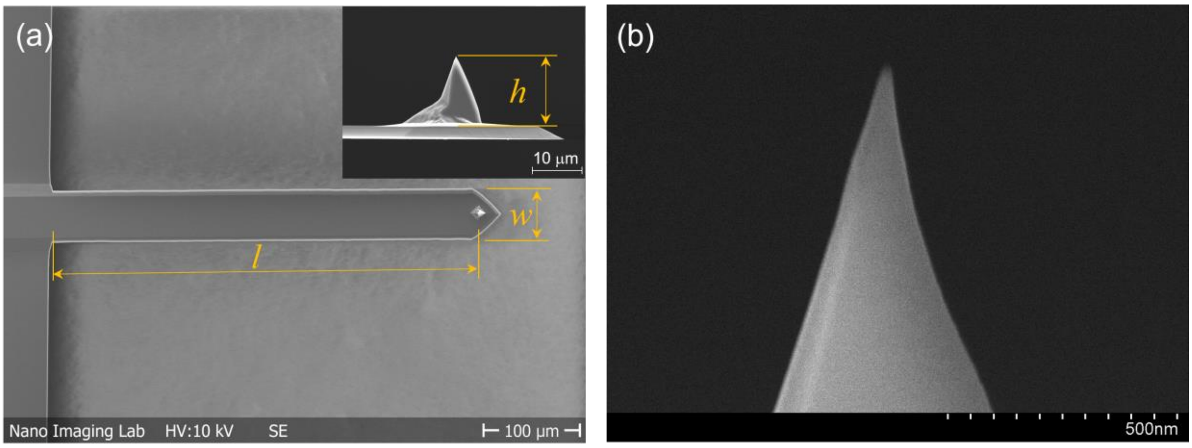

Supplementary Figure 2. SEM characterization of the AFM probe. (a) Dimensions of the cantilever and the height of the tip. (b) Zoom-in image of the tip.

Supplementary Note 3. Determination of Grain Boundary Angle

The GB angle θ_{GB}, i.e., the relative orientation configuration between two neighboring grains, is determined by fast Fourier transform (FFT) analysis of the atomic structures of the PolyGr layer provided by high-resolution characterization images (see, e.g., Supplementary Figure 3). The GB misfit angle, θ_{GB}, is obtained by measuring the angle between the two rotated FFT patterns (see white arrows in Supplementary Figure 3b), yielding, in this case, $\theta_{GB} = 21.43 \pm 0.66°$. The inner bright area in the FFT image is related to the long-range moiré superstructures.

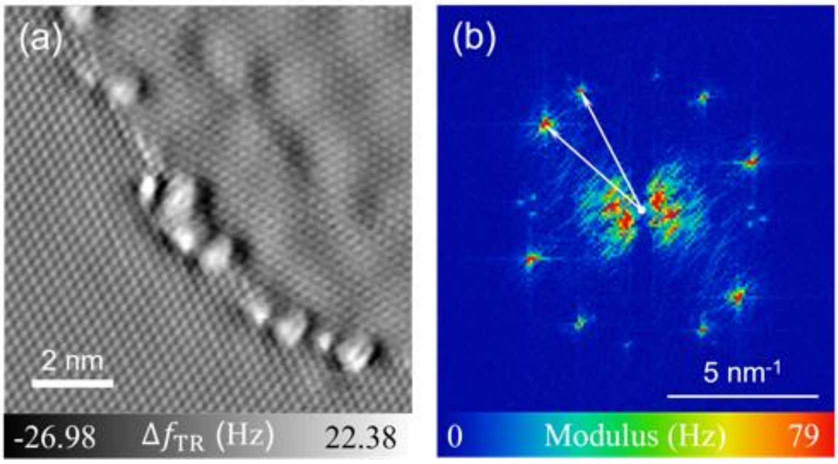

Supplementary Figure 3. Characterization of the misfit angle of the GB appearing in Fig. 1c of the main text. (a) High-resolution NC-AFM image of the moiré and atomic structure near a GB of a PolyGr surface using the torsional frequency shift Δf_{TR} mode (same as Fig. 1c of the main text). (b) FFT of panel a, giving a GB misfit angle of $\theta_{GB} = 21.43 \pm 0.66°$.

In addition, GB misfit angles have been determined independently using atomic resolution contact AFM lateral force maps and high-resolution low-temperature ($< 5K$) scanning tunneling microscope (LT-STM) topography images. Supplementary Figure 4a presents such a high-resolution lateral force map for the GB appearing in Fig. 2a of the main text, the misfit angle of which is determined via FFT analysis (Supplementary Figure 4b) to be $\theta_{GB} = 2.35 \pm 0.10°$.

For small angle GBs ($\theta_{GB} < 10°$) with discrete dislocations, each GB dislocation can form an individual upward protrusion.[2] This allows us to validate the FFT determination of the GB misfit

angle by comparing the extracted θ_{GB} values to those obtained independently from the periodic distance, *D*, between neighboring GB protrusions, using Frank's equation:[3,4]

$$\theta_{\mathrm{GB}} = 2\arcsin\frac{|\vec{b}_{(1,0)}|}{2D}. \qquad \text{(S1)}$$

Here, $\vec{b}_{(1,0)}$ is the Burgers vector of the most common edge-sharing heptagon-pentagon pair dislocation in graphene ($|\vec{b}_{(1,0)}| = 2.46$ Å, see Supplementary Figure 5a). For the GB appearing in Supplementary Figure 4a, periodic features (e.g., grey dots on the left of the GB) separated by $D = 5.34 \pm 0.23$ nm are observed. Substituting this value into Eq. S1, we obtain a theoretical estimation of the GB angle of $\theta_{\mathrm{GB}} \approx 2.64°$, in good agreement with our FFT analysis value of $\theta_{\mathrm{GB}} = 2.35 \pm 0.10°$.

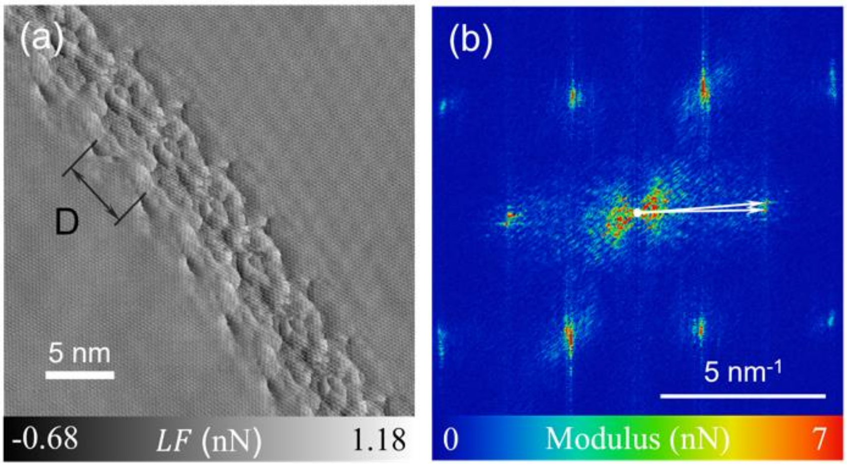

Supplementary Figure 4. Characterization of the misfit angle of the GB appearing in Fig. 2a of the main text. (a) High-resolution lateral force map of the GB region. The averaged distance between neighboring dislocations is $D = 5.34 \pm 0.23$ nm, yielding a misfit angle of $\theta_{\mathrm{GB}} \approx 2.64°$, via Eq. S1. (b) Corresponding FFT analysis of panel a, giving a GB angle of $\theta_{\mathrm{GB}} = 2.35 \pm 0.10°$.

The summarized experimental results of D vs. θ_{GB} (obtained via FFT analysis) as well as the corresponding predictions from Eq. S1 are presented in Supplementary Figure 5b. The good agreement between the experimental results and the theoretical predictions from Eq. S1, especially in the low GB misfit angle regime, further validates our experimental determination of the GB misfit angle. Furthermore, it also suggests that the GBs obtained in this study are mainly of (1,0) type (see

Supplementary Figure 5a) heptagon-pentagon pair dislocation, the Burgers vector of which was used for the determination of the misfit angle via Eq. S1.

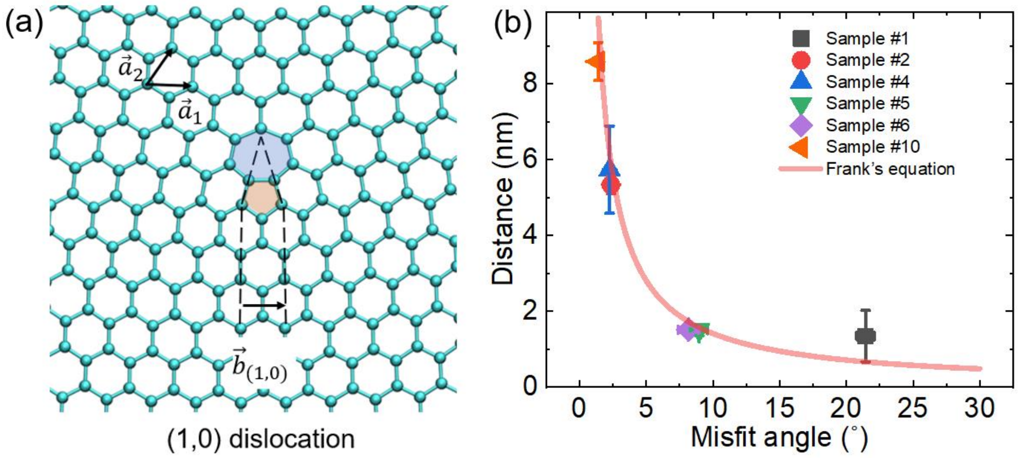

Supplementary Figure 5. Relation between GB misfit angle and inter-protrusion distance. (a) Illustration of the (1,0) type pentagon-heptagon dislocation and its corresponding Burgers vector. (b) Inter-protrusion distance dependence on the GB misfit angle as obtained using FFT analysis of high-resolution topography maps (symbols) compared to the theoretical prediction of Frank's equation (red line, Eq. S1). Error bars represent the corresponding standard deviation obtained by performing at least 3 to 5 independent measurements.

Supplementary Note 4. Characterization of Additional Grain Boundaries

In the main text, we presented the analysis of two GBs. Here, we provide LT-STM results for additional GBs of different misfit angles. The experiments were carried out using a commercial low temperature (~5 K) qPlus STM/AFM instrument (Omicron GmbH) operated with Nanonis RC5 electronics, which prevents thermal drifts. The polycrystalline graphene samples were transferred from the room-temperature UHV AFM apparatus into the low-temperature STM/AFM system via an UHV transfer suitcase (Ferrovac, base pressure of $\leq 5\times10^{-10}$ mbar). Before the STM measurement, a soft annealing process was conducted at a temperature of 400 K for 10 min to remove potential contaminants. All STM images were acquired in the constant-current mode. Based on FFT analysis of the LT-STM topography images, we identified a series of GB angles in the range of 2−28° (see Supplementary Figures 6-10).

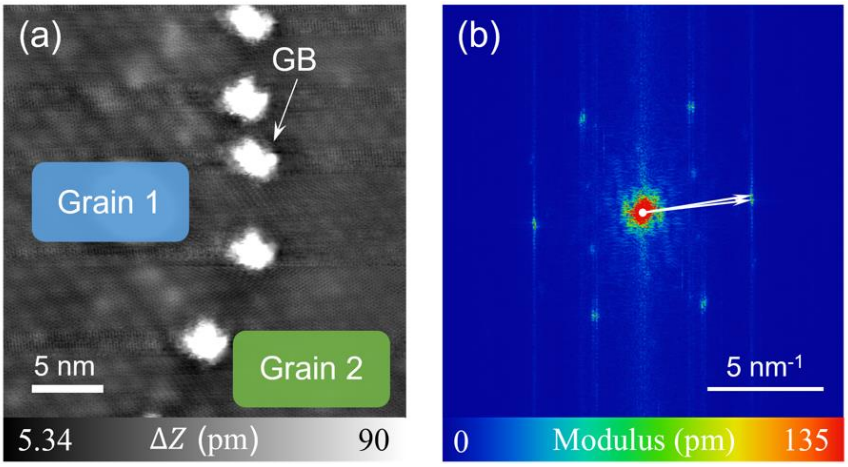

Supplementary Figure 6. Characterization of a GB with misfit angle of $\theta_{\mathrm{GB}} = 2.25 \pm 0.09^{\circ}$. (a) High resolution LT-STM image showing the moiré and the atomic structure on both sides of a GB. The average distance between neighboring GB protrusions is $D = 5.74 \pm 1.15$ nm. (b) The corresponding FFT map. The white arrows mark the two rotated FFT patterns associated with the atomic lattices of the two grains. Measurement parameters: $V_{\mathrm{tip}} = 3$ mV and $I = 1$ pA.

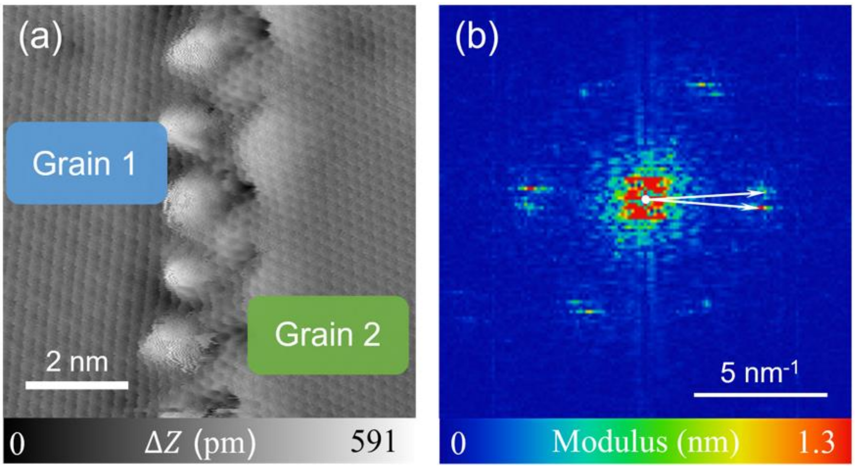

Supplementary Figure 7. Characterization of a GB with misfit angle of $\theta_{\mathrm{GB}} = 8.15 \pm 0.76^{\circ}$. (a) High resolution LT-STM image showing the moiré and the atomic structure on both sides of a GB. The average distance between neighboring GB protrusions is $D = 1.52 \pm 0.12$ nm. (b) The corresponding FFT map. The white arrows mark the two rotated FFT patterns associated with the atomic lattices of the two grains. Measurement parameters: $V_{\mathrm{tip}} = 12$ mV and $I = 50$ pA.

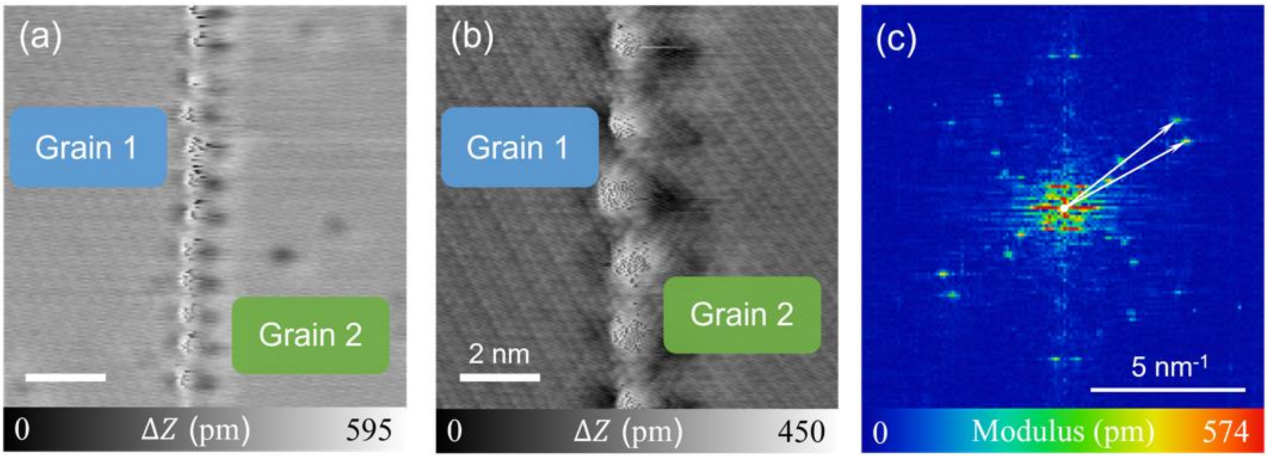

Supplementary Figure 8. Characterization of a GB with misfit angle of $\theta_{\mathrm{GB}} = 8.96 \pm 0.63^{\circ}$. (a) LT-STM image showing the moiré superlattices at the two grain regions. (b) High resolution LT-STM image showing the moiré and the atomic structure on both sides of the same GB. The average distance between neighboring GB protrusions is $D = 1.51 \pm 0.15$ nm. (c) The corresponding FFT map. The white arrows mark the two rotated FFT patterns associated with the atomic lattices of the two grains. Measurement parameters: $V_{\mathrm{tip}} = 8$ mV and $I = 50$ pA.

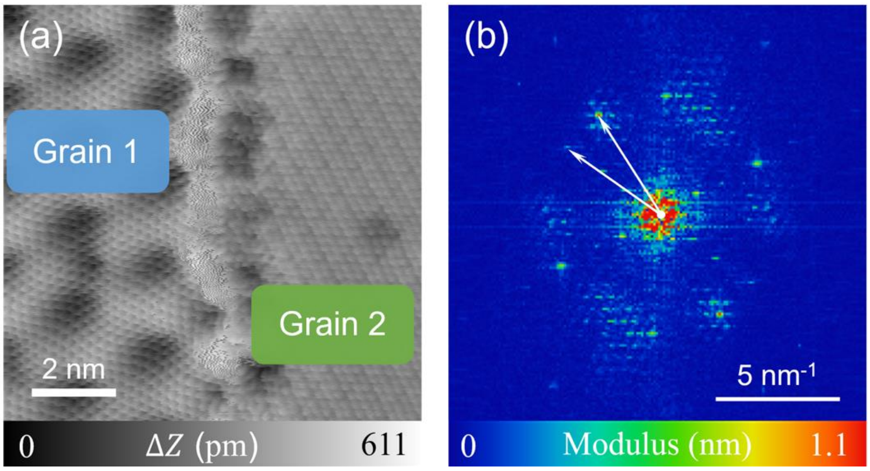

Supplementary Figure 9. Characterization of a GB with misfit angle of $\theta_{GB} = 21.94 \pm 0.78^{\circ}$. (a) High resolution LT-STM image showing the moiré and the atomic structure on both sides of a GB. (b) The corresponding FFT map. The white arrows mark the two rotated FFT patterns associated with the atomic lattices of the two grains. Measurement parameters: $V_{tip} = 8$ mV and $I = 50$ pA.

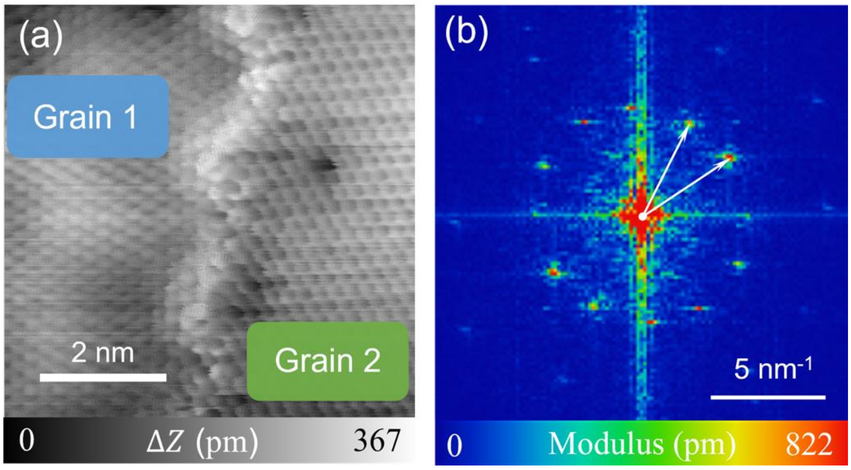

Supplementary Figure 10. Characterization of a GB with misfit angle of $\theta_{GB} = 27.31 \pm 0.75^{\circ}$. (a) High resolution LT-STM image showing the moiré and the atomic structure on both sides of a GB. (b) The corresponding FFT map. The white arrows mark the two rotated FFT patterns associated with the atomic lattices of the two grains. Measurement parameters: $V_{tip} = 28$ mV and $I = 50$ pA.

Supplementary Note 5. Energy Dissipation Route and Average Friction Calculation

In addition to the atomic scale topography characterization, the averaged lateral force map (obtained as half of the difference between the lateral forces measured at each location during forward and backward scanning) shows higher friction patterns along at the GB region (see Supplementary Figure 11). These patterns match the topography features, indicating that the major contribution to the energy dissipation originates from the upward protrusions of corrugated graphene GB. Since these are the dominating friction contributions, the inclusion of bulk grain areas in the average friction calculation over the entire square domain presented has a minor effect on our conclusions of the GB frictional behavior.

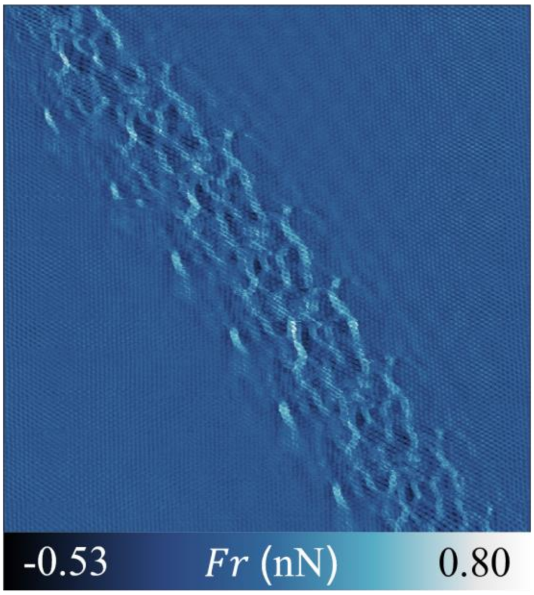

Supplementary Figure 11. Averaged friction force map measured at the GB region presented in Fig. 2 of main text.

Supplementary Note 6. Reproducibility and Additional Grain Boundary Friction Measurements

In the main text, we presented results of frictional measurements on a corrugated GB of misfit angle $\theta_{\mathrm{GB}} = 2.35 \pm 0.10^{\circ}$, demonstrating unconventional negative friction coefficients (NFCs) and non-monotonic velocity dependence. To verify the reproducibility of these observations, we repeated our measurements on a different corrugated GB with misfit angle of $\theta_{\mathrm{GB}} = 14.42 \pm 0.47^{\circ}$ (Supplementary Figure 12a). As shown in Supplementary Figures 12b-d the typical lateral force loops measured at different normal loads, reveal that frictional energy dissipation emerges mainly over the GB region, showing a significant reduction with increasing normal load, similar to Fig. 2c-e of the main text. Furthermore, both the negative slope of the load dependence and the non-monotonic velocity dependence of the measured friction for this system are fully reproduced, in agreement with the results presented in Fig. 2f-g of the main text. Similar NFC behavior is observed also for a $\theta_{\mathrm{GB}} = 1.39 \pm 0.11^{\circ}$ corrugated GB (see Supplementary Figure 13). Therefore, we conclude that the revealed frictional properties of corrugated GBs are of general nature and not limited to a specific system.

As a separate validation test, we present in Supplementary Figure 14 five independent measurement repetitions of the friction force velocity dependence over the $\theta_{\mathrm{GB}} = 2.35 \pm 0.10°$ corrugated GB. The results demonstrate a relatively narrow spread thus signifying the reproducibility and robustness of our GB friction measurements.

To validate that the non-monotonic velocity dependence of the friction force is associated with the negative differential friction coefficient region, we repeated those measurements for the $\theta_{\mathrm{GB}} = 2.35 \pm 0.10°$ under a normal load of 5 nN, corresponding to the plateau region in Fig. 2f. Supplementary Figure 15, demonstrates clear monotonic logarithmic increase of the friction force with sliding velocity in this region. We note that when comparing the friction force measured at a sliding velocity of 41.9 $\mathrm{nm} \cdot \mathrm{s}^{-1}$ and a normal load of 5 nN to the corresponding value appearing in Fig. 2f, we obtain a somewhat lower value (by ~ 2 pN) for the former. This may be attributed to probing different GB regions in different measurements. Similar minor deviations are also observed between panels of Fig. 2f and 2g in the main text.

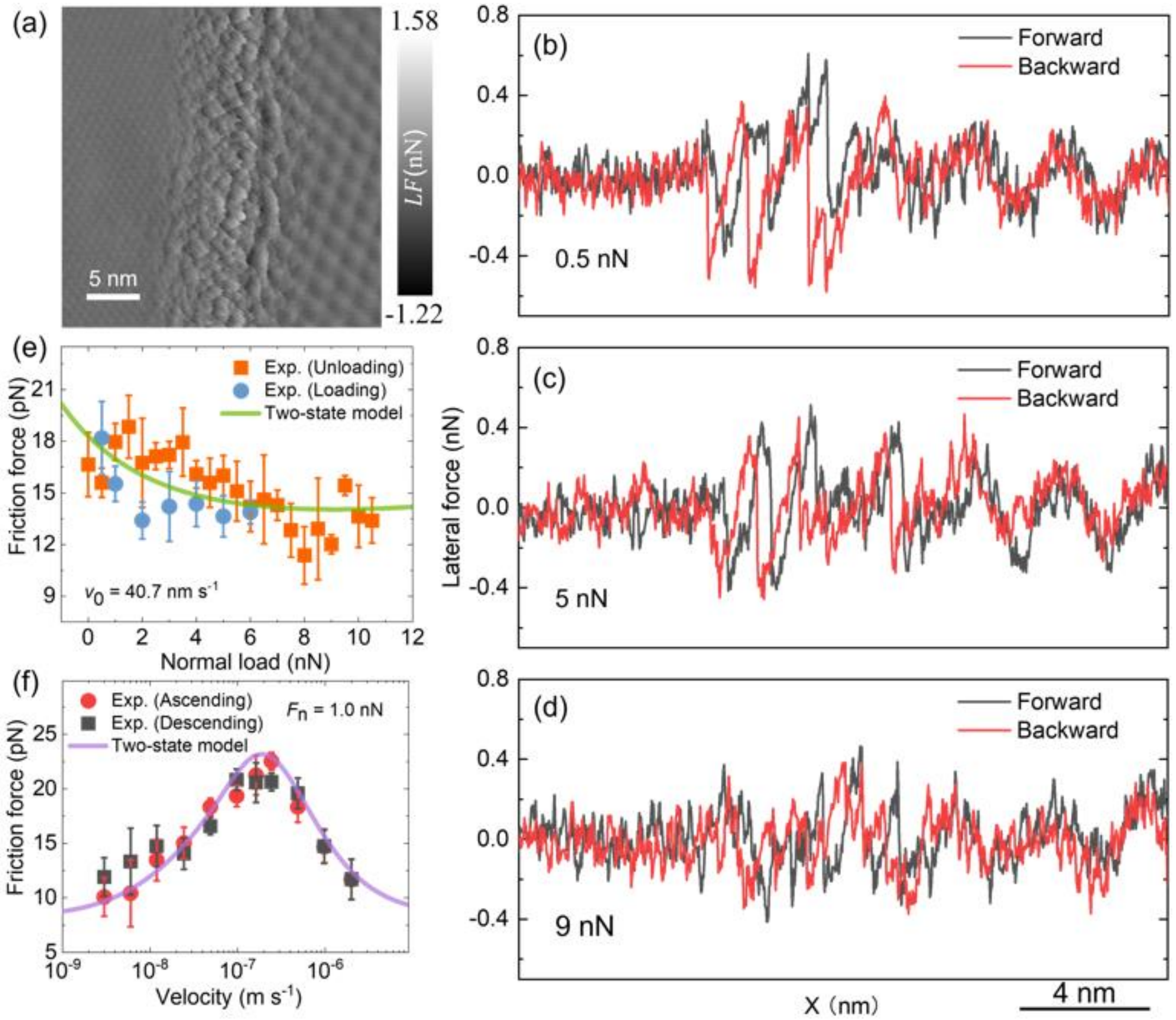

Supplementary Figure 12. Frictional measurements reproducibility. (a) Lateral force map for the $\theta_{\rm GB} = 14.42 \pm 0.47^{\circ}$ corrugated GB, measured under a normal load of 5.7 nN and a sliding velocity of $292.9\ \mathrm{nm} \cdot \mathrm{s}^{-1}$. (b)-(d) Forward (black) and backwards (red) lateral force traces measured under low (0.5 nN), medium (5 nN), and high (9 nN) normal loads, respectively. (e) Load and (f) velocity dependence of the measured friction force between the sliding AFM tip and the same graphene GB. Error bars represent the corresponding standard deviation obtained by performing at least 5 to 6 independent scans. The fitting parameters of the phenomenological model in panels b (green line) and c (purple line) are: $T = 300$ K, $E_1 = 0.13$ eV, $E_2 = 0.18$ eV, $\Delta x = 10.8$ Å, $\alpha = 0.1\ \mathrm{eV} \cdot \mathrm{GPa}^{-1}$, $\beta = 0.2$, $c_0 = 0.05$ eV, $N = 2$, $f_0 = 3.26$ kHz, $\mu = 3 \times 10^{-4}$, $c_1 = 8$ pN. Here, the effective protrusion stiffness is calculated as $k_0 = \frac{E_1 + E_2}{\Delta x^2}$, reflecting the fact that the maximum elastic energy stored by the spring $\left(\frac{1}{2} k_0 \Delta x^2\right)$, cannot exceed $\Delta E_{\rm max}$ (see model description in the main text).

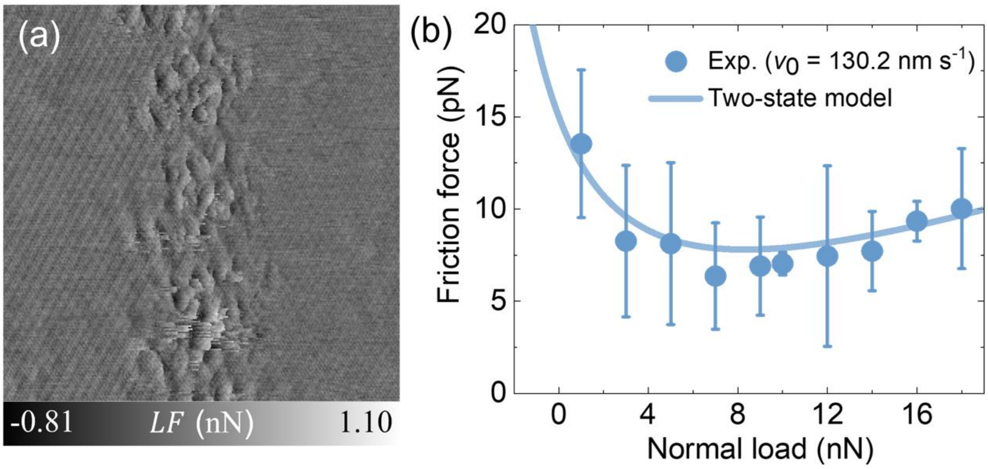

Supplementary Figure 13. Frictional load dependence measurement reproducibility. (a) Lateral force map for the $\theta_{\mathrm{GB}} = 1.39 \pm 0.11^{\circ}$ corrugated GB measured under a normal load of 7 nN and a sliding velocity of $130.2\ \mathrm{nm} \cdot \mathrm{s}^{-1}$. (b) Load dependence of the measured friction force between the sliding AFM tip and the same graphene GB. Error bars represent the corresponding standard deviation obtained by performing 3 independent scans. The fitting parameters of the phenomenological model in panels b (blue line) are: $T = 300$ K, $E_1 = 0.22$ eV, $E_2 = 0.32$ eV, $\Delta x = 10.8$ Å, $\alpha = 0.2\ \mathrm{eV} \cdot \mathrm{GPa}^{-1}$, $\beta = 0.3$, $c_0 = 0.1$ eV, $N = 1$, $f_0 = 39.06$ kHz, $\mu = 4 \times 10^{-4}$, $c_1 = 1$ pN.

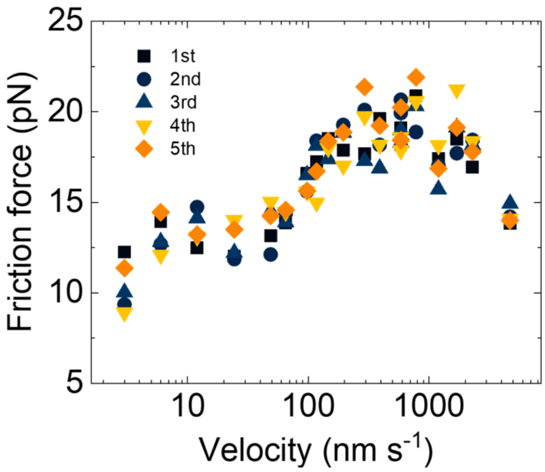

Supplementary Figure 14. Frictional velocity dependence measurement reproducibility. Five independent measurement repetitions are performed on the $\theta_{\mathrm{GB}} = 2.35 \pm 0.10^{\circ}$ corrugated GB. The average and standard deviation of these results are presented in Fig. 2g of the main text.

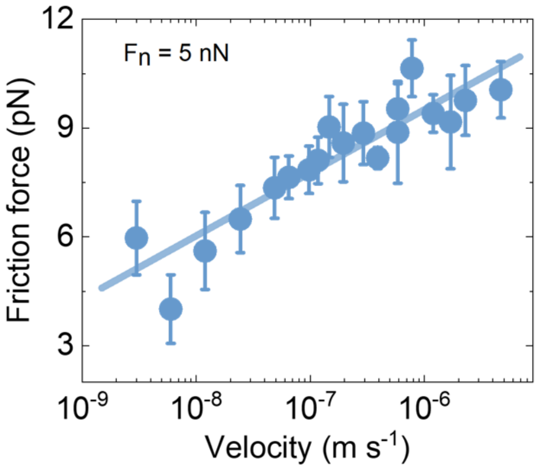

Supplementary Figure 15. Velocity dependence of the measured friction force between the sliding AFM tip and the $\theta_{\mathrm{GB}} = 2.35 \pm 0.10°$ GB presented in Fig. 2 of main text under a normal load of 5 nN, corresponding to the friction plateau regime of Fig. 2f of main text. Error bars represent the corresponding standard deviation obtained by performing 5 independent scans. The solid line represents logarithmic fitting as a guide for the eye.

Supplementary Note 7. Molecular Dynamics Simulations

7.1 Simulation setup and protocol

The simulation protocol used in the present study is similar to that used in Ref. 5. The non-reactive molecular dynamics (MD) simulation model system consists of a spherical-cap shaped diamond tip (2.3 nm in height, 5 nm in radius at the cut surface) sliding atop a PolyGr layer supported by a 1.36 nm thick Pt(111) substrate, as shown in Supplementary Figure 16a. Note that we use a diamond tip model rather than silicon, as used in the experiment, due to the structural simplicity of the former. Furthermore, the use of non-reactive dynamics, which excludes tip-substrate covalent bonding, is justified by the fact that our simulation results correctly describe both the force traces and the experimentally observed dependence of friction on the normal load. Since the frictional energy dissipation mechanism of corrugated GBs relies on intrinsic protrusion buckling/unbuckling processes, the specific identity of the tip is expected to have a minor effect on the results. To rationalize the experimental results, we generate two PolyGr model systems with GB misfit angles of $\theta_{\mathrm{GB}} = 2°$ and 27.8°, matching the experimentally studied corrugated GBs. To that end, we use a Voronoi tessellation method developed by Shekhawat et al.[6,7] The lateral dimensions of the PolyGr layers with $\theta_{\mathrm{GB}} = 2°$ and 27.8° are 29.4×28.1 and 30.0×27.7 nm^2, respectively. The PolyGr layers are then placed on a laterally periodic Pt(111) substrate of lateral dimensions of 41.6×40.8 nm^2 and thickness of 1.13 nm with the bottom layer atoms fixed at their positions, such that the left grain is aligned with the lattice direction of the Pt(111) surface, leading to moiré superstructures period of ~2.2 nm. Correspondingly, the superstructure periods of the right grain are ~2.0 nm for a GB misfit angle is $\theta_{\mathrm{GB}} = 2°$, and close to the atomic lattice period for $\theta_{\mathrm{GB}} =$27.8°, consistent with the experimental configurations shown in Figs. 2a and 3a of the main text.

Prior to the dynamical simulations, the PolyGr/Pt(111) system is annealed at a temperature of 1,000 K, then the spherical-cap shaped diamond tip is placed on the left grain at a lateral distance of ~7 nm from the GB and at a height of 3.3 Å. To maintain the shape of the tip, the top two layers of the spherical-cap are kept rigid, while the rest tip atoms are unconstrained (see Supplementary Figure 16b). A dummy atom mimicking a moving stage is coupled to the center of mass of the rigid section of the tip via a lateral spring with stiffness of $10\ \mathrm{N \cdot m^{-1}}$. The geometry of the combined system is then further relaxed under an external normal load (the same load used in the corresponding dynamic simulation) using the FIRE algorithm[8,9] with a convergence force criterion of $10^{-4}\ \mathrm{eV \cdot Å^{-1}}$. The normal load is enforced by applying a vertical constant force on each of the atoms in the rigid section

of the tip, with magnitude ranging from 0 to 0.01 nN per atom, corresponding to an overall normal load in the range of 0 to 24.4 nN.

The zero-temperature dynamic simulations are performed by driving the diamond tip with the dummy atom moving at a constant velocity of $v_0 = 2\ \mathrm{m \cdot s^{-1}}$ along the x lateral direction. To remove the generated heat, viscous velocity damping with a damping coefficient of $\eta = 1.0$ ps^{-1} is applied to flexible regions of the tip and the Pt(111) substrate far away from the sliding interface (see Supplementary Figure 16b). To that end, damping forces $\mathbf{f}^i_{\mathrm{damp,tip}}(t)$ and $\mathbf{f}^i_{\mathrm{damp,Pt}}(t)$ are applied to each atom, i, within the damped regions in the tip and the Pt substrate, respectively, via:

$$\begin{cases} \mathbf{f}^i_{\mathrm{damp,tip}}(t) = -m_{\mathrm{C}}\eta\left(v^i_x(t) - v_0\right)\hat{\mathbf{x}} - m_{\mathrm{C}}\eta v^i_y(t)\hat{\mathbf{y}} - m_{\mathrm{C}}\eta v^i_z(t)\hat{\mathbf{z}} \\ \mathbf{f}^i_{\mathrm{damp,Pt}}(t) = -m_{\mathrm{Pt}}\sum_{\alpha=x,y,z}\eta v^i_\alpha(t)\,\hat{\boldsymbol{\alpha}} \end{cases} \tag{S2}$$

Here, m_{C} and m_{Pt} are the atomic masses of carbon and Pt, respectively, $v^i_\alpha(t)$ is the α Cartesian velocity component of the damped i^{th} atom at time t, and $\hat{\boldsymbol{\alpha}} = \hat{\mathbf{x}}, \hat{\mathbf{y}}, \hat{\mathbf{z}}$ are unit vectors along the Cartesian x, y and z directions, respectively. Note that the damping in the sliding (x) direction in the tip is applied to the relative velocity with respect to the moving stage, accounting for the fact that in realistic scenarios viscous dissipation is caused by the internal degrees of freedom of the sheared bodies. We verify that our choice of damping coefficient has minor effect on the dynamics at the shear interface.[2]

To prevent global sliding and/or rotation of the PolyGr layer due to the tip motion, two ribbons of outskirt carbon atoms at the two parallel sides in the PolyGr layer (see stripes regions in Supplementary Figure 16) are constrained to their initial position via lateral springs of stiffness $0.176\ \mathrm{N \cdot m^{-1}}$. For each normal load and GB angle, the dynamic simulation last for 7 ns, which allows us to eliminate initial transient effects. The lateral force traces are obtained by recording the instantaneous spring force exerted on the dummy atom along the sliding direction and inverting its sign. The average friction force is calculated by averaging the forward and backward force traces over distances of 6.5 and 6 nm, spanning the $\theta_{\mathrm{GB}} = 2°$ and 27.8° GBs, respectively, as shown in Supplementary Figure 17.

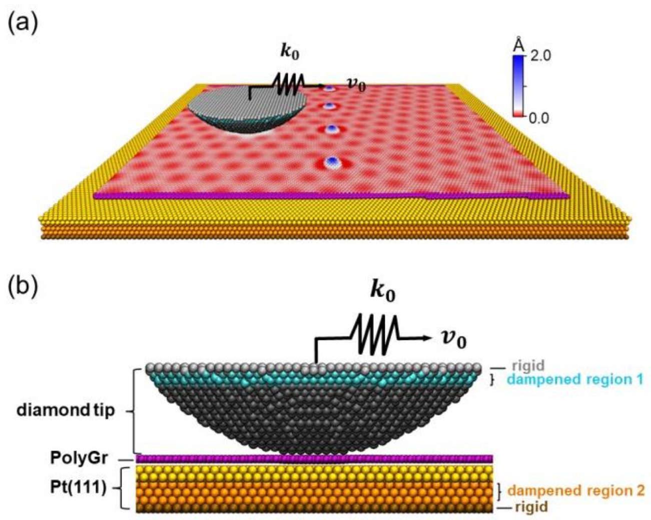

Supplementary Figure 16. MD simulation setup. (a) Perspective view of the simulation model system with $\theta_{GB} = 2°$. (b) Side view of the tip region presented in panel (a). Brown, orange, and yellow spheres in the substrate region represent rigid, dampened, and flexible Pt atoms. Light-gray, cyan, and dark-gray spheres in the tip region represent rigid, dampened, and flexible carbon atoms. The unconstrained graphene layer atoms are color coded according to their out-of-plane corrugation (see color bar in panel (a)), and the constrained graphene atoms are represented by purple spheres.

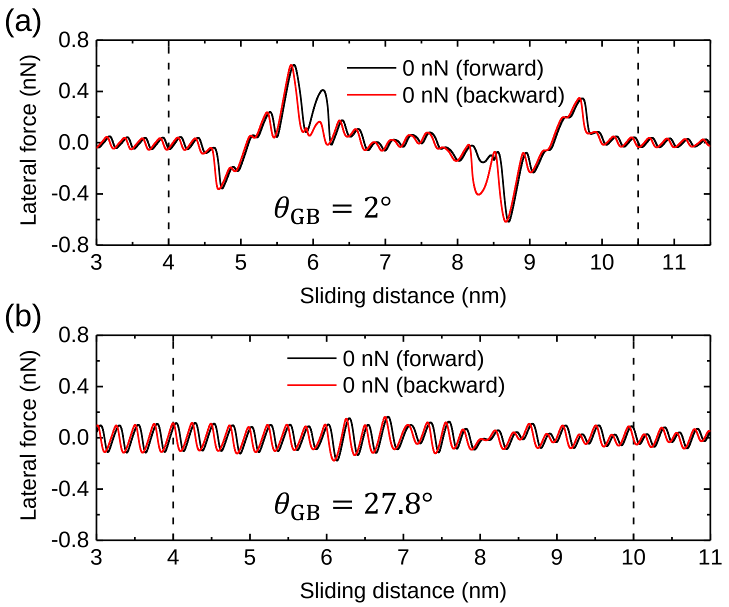

Supplementary Figure 17. Lateral forward (black) and backward (red) force traces as a function of sliding distance over the (a) $\theta_{GB} = 2°$ and (b) $\theta_{GB} = 27.8°$ GBs under zero normal load. The region between the dashed vertical lines is used to calculate the average friction force.

7.2 Additional simulation results

Complementary to the simulation trajectories shown in Fig. 4e of the main text, we present in Supplementary Figure 18 additional trajectories of atomic height and vertical velocity for a chosen atom at a dislocation of a $\theta_{\mathrm{GB}} = 2°$ GB. The results demonstrate that as normal load increases, the instantaneous buckling velocity decreases until the trajectories become smooth. Consequently, the buckling induced energy dissipation reduces with increasing normal load.

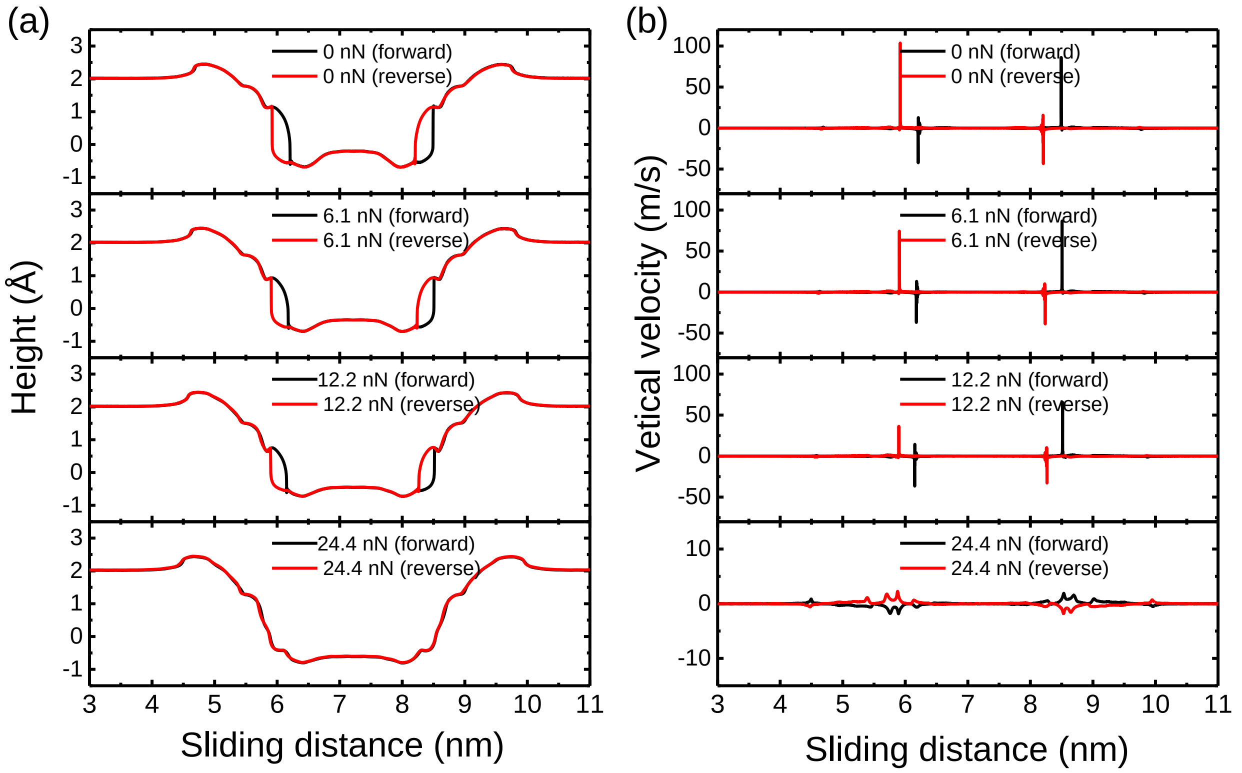

Supplementary Figure 18. Additional simulation trajectories of (a) atomic height and (b) vertical velocity, under different normal loads, of an atom residing at a dislocation of a $\theta_{\mathrm{GB}} = 2°$ GB.

7.3 Simulation parameters for carbon-Pt interactions

In view of the lack of anisotropic potentials for carbon-Pt interactions, we use the Lennard-Jones (LJ) potential, which was shown to provide satisfactory qualitative interpretation of complex frictional mechanisms of graphene on various substrates.[5,10-12] Our choice of LJ parameters is primarily based on benchmarking the binding energy ΔE_{b} and equilibrium distance d against available experimental and computational (using density functional theory (DFT)) reference data. Experimental and DFT data for the graphene/Pt(111) interface are summarized in Supplementary

Table 1, along with previous structural relaxation results based on the LJ potential. Most previous DFT calculations predict adsorption energies in the range of $\Delta E_{\mathrm{b}} = 30 - 90\ \mathrm{meV} \cdot \mathrm{C}^{-1}$,[13-16] and equilibrium distances of 3.1-3.8 Å. Experimental data suggest an equilibrium graphene/Pt(111) distance of 3.3 Å.[17]

We note that several studies using the LJ potential for the graphene/Pt interface overestimated the binding energy and underestimated the equillibrium distance with respect to DFT and experimental reference data.[18,19] Moseler et al.[10] used the following set of LJ parameters: $\varepsilon_{\mathrm{C-Pt}} = 0.011236$ eV and $\sigma_{\mathrm{C-Pt}} = 3.408$ Å, fitted against the DFT reference of $\Delta E_{\mathrm{b}} = 68\ \mathrm{meV} \cdot \mathrm{C}^{-1}$ [14] and the experimental reference of $d = 3.3$ Å[17] with a LJ cutoff distance of $R_{\mathrm{cut}} = 6$ Å. The latter, however, may be too small to fully capture long-range van der Waals contributions. Hence, in the present study, we adopt the following alternative set of parameters: $\varepsilon_{\mathrm{C-Pt}} = 0.006$ eV and $\sigma_{\mathrm{C-Pt}} = 3.35$ Å with a LJ cutoff distance $R_{\mathrm{cut}} = 12$ Å. This parameterization provides a binding energy of $\Delta E_{\mathrm{b}} = 48.4\ \mathrm{meV} \cdot \mathrm{C}^{-1}$ and an equilibrium distance $d = 3.3$ Å, in good agreement with vdW-DF and vdW-DF2 results[14,16] and the experimental equilibrium distance.[17] Furthermore, the moiré superstructure periodicity and corrugation obtained using this force-field (see Figs. 4a-b of the main text) are compatible with our experimental results and with previous experimental observations.[15] This supports the suitability of our LJ potential parameterization for studying the frictional behavior of PolyGr layers deposited atop the Pt(111) surface.

Supplementary Table 1. Binding energies (ΔE_b) and equilibrium distances (d) obtained for the graphene/Pt(111) interface via DFT and classical Lennard-Jones force-field calculations. For the latter, the corresponding potential parameters are provided. An available experimental result for the graphene/Pt(111) equilibrium distance is provided for comparison.

Reference	$\varepsilon_{\mathrm{C-Pt}}$ (eV)	$\sigma_{\mathrm{C-Pt}}$(Å)	$\Delta E_{\mathrm{b}}(\mathrm{meV \cdot C^{-1}})$	d(Å)	R_{cut}(Å)	Method
Khomyakov et al. (2009)[13]			38	3.3		DFT(LDA)
Hamada & Otani (2010)[14]			52	3.8		DFT(vdW-DF)
			54	3.71		DFT(vdW-DF2)
			89	3.17		DFT(vdW-DF^{C09x})
			68	3.24		DFT(vdW-DF2^{C09x})
Gao et al. (2011)[15]			38.6-39.8	>3.1		DFT
Martínez et al.(2016)[20]			240-290	3.13-3.23		DFT(rPBE+D)
Mortensen et al. (2010)[16]			43	3.67		DFT(vdW-DF)
			33	3.25		DFT(LDA)
Sutter et al. (2009)[17]				3.3		Exp.
Morrow & Striolo (2007)[18]	0.02206	2.95	128	2.91	9	MD
Mi et al. (2020)[19]	0.4092	2.936	237	3.05 (2.894)[a]	9	MD
Moseler et al. (2014)[10]	0.011236	3.408	68 (66)[a]	3.3 (3.408)[a]	6	MD
			(88)[a]	(3.36)[a]	9	
			(94)[a]	(3.354)[a]	12	
This study	0.006	3.35	48.4	3.3	12	MD

[a] The values in parentheses are results of our calculations obtained using the LJ parameters provided in the cited studies with various R_{cut} values, as detailed in the table.

7.4 Effect of scanline direction

To further evaluate the dependence of friction force on the sliding direction, we performed additional MD simulations along an inclined scanline of 15° with respect to the GB normal. The force traces presented in Supplementary Figure 19a show significant energy dissipation at the GB, associated with strong buckling of GB protrusions and non-Amontons frictional behavior (friction reduction with increased normal load), as shown in Supplementary Figure 19b, both of which are consistent with the results obtained for the original scanline.

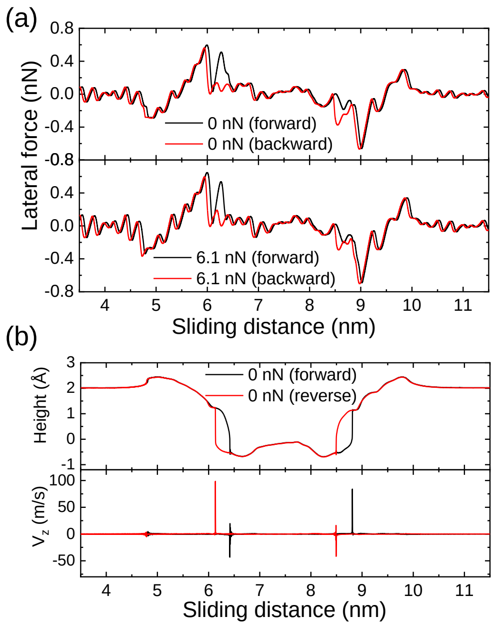

Supplementary Figure 19. MD simulation results for a θ_{GB} = 2° GB obtained along a 15° scanline direction with respect to the GB normal. (a) Lateral force trace loops obtained under normal loads of 0 and 6.1 nN; (b) GB atom height and velocity trajectories under zero normal load.

7.5 Effect of Pt substrate thickness

To check the effect of Pt slab thickness, we have repeated some of our simulations with a slab thickness of up to 13 nm, which is ~3 times the tip radius. Supplementary Figure 20 shows the load dependence of the average friction force obtained for various slab model thicknesses. Clearly, the results converge already for a slab thickness of 6.5 nm. For all substrate thicknesses considered, a similar nonmonotonic load dependence is obtained. For the 1.3 nm thick slab model, used to obtain the results presented in the main text, the minimum lies at a somewhat higher load and its value is somewhat larger. Nonetheless, the qualitative non-Amontons behavior remains the same.

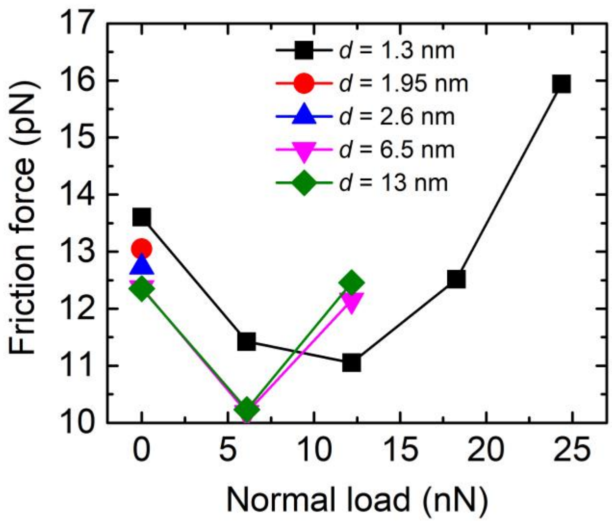

Supplementary Figure 20. Effect of Pt substrate model thickness on the load dependence of the average GB friction.

7.6 Contact profiles of graphene under different normal loads

In the main text, a constant contact area with a radius of 12 Å was assumed in the phenomenological two-state model. To justify the assumption of constant contact area under varying normal load we note that our experimental system involves a nanometric single crystal silicon AFM probe (PPP-CONT, Nanosensors), pointing in the <100> direction[21] and operating under relatively low normal load. Prior to the friction measurements, the native oxide had been etched away by Ar^+ sputtering, probably resulting in a stepped atomic structure at the tip apex. Under these conditions, the contact area is estimated to remain constant under loads below 12.53 nN,[22] which is higher than the upper bound of our experimentally applied load (<10 nN) for observing non-Amontons behavior. This is further supported by our atomistic MD simulation results that demonstrate a constant lateral deformation profile of the graphene surface under varying tip loads up to 12 nN (see Supplementary Figure 21).

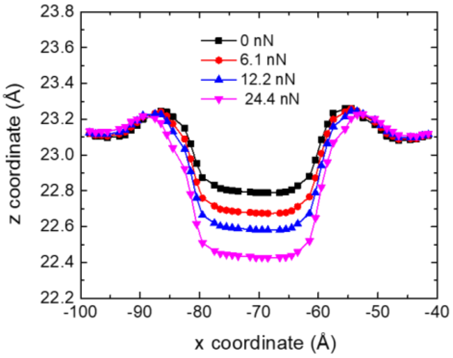

Supplementary Figure 21. MD simulation results of the deformation profiles of polycrystalline graphene grain under different tip normal loads obtained using a 1.3 nm thick Pt substrate.

Supplementary Note 8. Two-State Model Parameter Fitting

8.1 Extraction of the two-state model parameters from MD results and experiments

Whenever possible, the two-state model parameters have been extracted from the MD simulation results. Specifically, the bounds of bare buckling transition energy barriers ($E_1 = 0.18$ eV and $E_2 = 0.26$ eV), as well as the rate of reduction of the transition barrier with normal load (α = 0.2 eV $\cdot$ GPa^{-1}) were estimated from the instant kinetic energy pulses produced following GB buckling during the sliding simulations, as shown in Supplementary Figure 22. The characteristic sliding distance (Δx = 10.8 Å) is chosen to match the typical GB width (~1 nm). Assuming that the elastic energy stored in the effective spring (mimicking the GB in our phenomenological model) cannot exceed the transition energy barrier, we estimate the effective spring stiffness, k_0, via the relation $\frac{E_1+E_2}{2} = \frac{1}{2} k_0 \Delta x^2$. The value of the number of GB protrusions influenced by the tip, (N = 1 or 2) is calculated from the ratio between the tip radius and the inter-protrusion separation, D, and the attempt frequency, $f_0 = 16.76\,\mathrm{kHz}$, which corresponds to the characteristic frequency of the cantilever, is chosen to be consistent with previous experimental values.[23] The remaining parameters ($\beta = 0.2$, $c_0 = 0.05$ eV, $\mu = 6 \times 10^{-4}$ and $c_1 = 4.5$ pN) are fitted to obtain good agreement between the model and the experimental and simulation results.

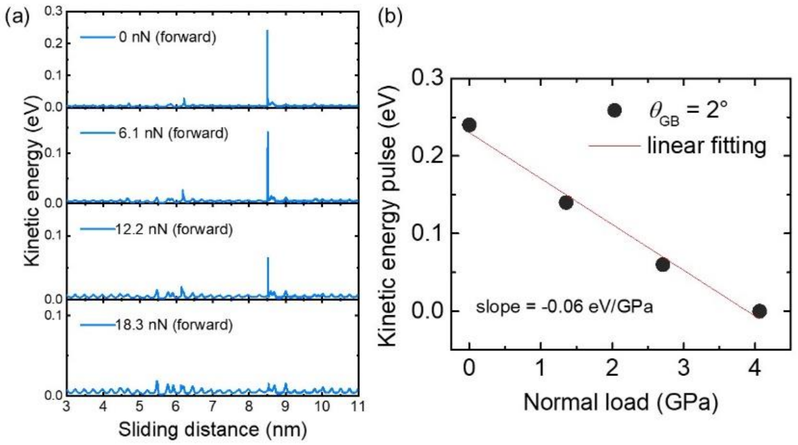

Supplementary Figure 22. (a) Kinetic energy profiles calculated for a $\theta_{GB} = 2°$ GB system under various normal loads. (b) Kinetic energy pulse intensity as a function of normal load.

8.2 Comparison between the two-state model and MD simulation results

To demonstrate the agreement between the refitted two-state model and the zero-temperature simulation results, presented in Fig. 4g of the main text, we modified Eq. 4 of the main text for the energy dissipation to account for zero temperature conditions.[24] The resulting equation reads as:

$$\Delta w(\sigma, \Delta E_{\max}) = \frac{k_0}{2} x_1^2 \left[1 - H\big(\Delta E_{\min}(\sigma, \Delta x)\big)\right], \tag{S3}$$

where $x = x_1$ is the location at which $\Delta E(x_1, \sigma) = -\frac{\Delta E_{\max} - \Delta E_{\min}(\sigma, \Delta x)}{\Delta x} x_1 + \Delta E_{\max} = 0$, yielding:

$$x_1 = \frac{\Delta E_{\max} \Delta x}{(1-\beta)\Delta E_{\max} + c_0 + \alpha\sigma}. \tag{S4}$$

The resulting comparison is given in Supplementary Figure 23 showing a similar qualitative behavior for the atomistic simulations and the parametrized zero Kelvin two-state model, with a vertical shift that may be attributed to the use of a simplified tip model in the atomistic simulations.

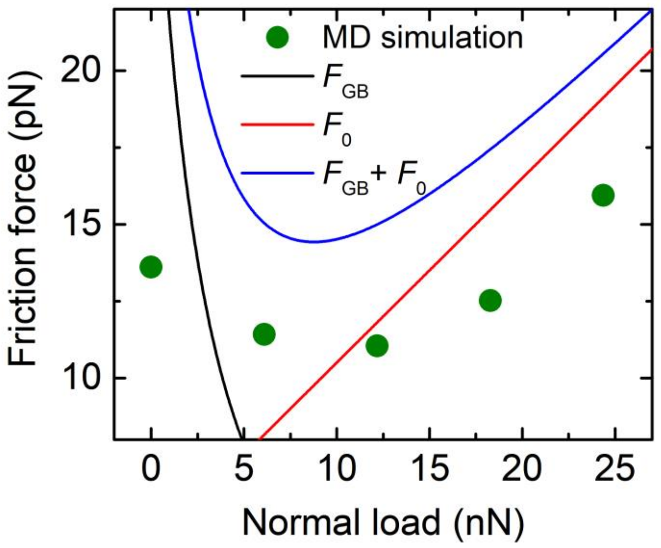

Supplementary Figure 23. Comparison between the load dependence of the friction force obtained using MD simulations (green circles) and the two-state model (blue line) for the θ_{GB} = 2° system. The GB (black line) and pristine surface (red line) contributions to the two-state model are also presented.

Supplementary References:

1 Meyer, E., Bennewitz, R. & Hug, H. J. *Scanning Probe Microscopy: The Lab on a Tip* Graduate Texts in Physics, (Springer Cham, 2021).

2 Gao, X., Ouyang, W., Hod, O. & Urbakh, M. Mechanisms of frictional energy dissipation at graphene grain boundaries. *Phys. Rev. B* **103**, 045418 (2021).

3 Anderson, P. M., Hirth, J. P. & Lothe, J. *Theory of dislocations*, (Cambridge University Press, 2017).

4 Yazyev, O. V. & Louie, S. G. Topological defects in graphene: Dislocations and grain boundaries. *Phys. Rev. B* **81**, 195420 (2010).

5 Song, Y., Gao, X., Hinaut, A., Scherb, S., Huang, S., Glatzel, T., Hod, O., Urbakh, M. & Meyer, E. Velocity Dependence of Moiré Friction. *Nano Lett.* **22**, 9529-9536 (2022).

6 Ophus, C., Shekhawat, A., Rasool, H. & Zettl, A. Large-scale experimental and theoretical study of graphene grain boundary structures. *Phys. Rev. B* **92**, 205402 (2015).

7 Shekhawat, A. & Ritchie, R. O. Toughness and strength of nanocrystalline graphene. *Nat. Commun.* **7**, 10546 (2016).

8 Bitzek, E., Koskinen, P., Gähler, F., Moseler, M. & Gumbsch, P. Structural Relaxation Made Simple. *Phys. Rev. Lett.* **97**, 170201 (2006).

9 Guénolé, J., Nöhring, W. G., Vaid, A., Houllé, F., Xie, Z., Prakash, A. & Bitzek, E. Assessment and optimization of the fast inertial relaxation engine (fire) for energy minimization in atomistic simulations and its implementation in lammps. *Comput. Mater. Sci.* **175**, 109584 (2020).

10 Klemenz, A., Pastewka, L., Balakrishna, S. G., Caron, A., Bennewitz, R. & Moseler, M. Atomic Scale Mechanisms of Friction Reduction and Wear Protection by Graphene. *Nano Lett.* **14**, 7145-7152 (2014).

11 Liu, Z., Vilhena, J. G., Hinaut, A., Scherb, S., Luo, F., Zhang, J., Glatzel, T., Gnecco, E. & Meyer, E. Moire-Tile Manipulation-Induced Friction Switch of Graphene on a Platinum Surface. *Nano Lett.* **23**, 4693-4697 (2023).

12 Li, S., Li, Q., Carpick, R. W., Gumbsch, P., Liu, X. Z., Ding, X., Sun, J. & Li, J. The evolving quality of frictional contact with graphene. *Nature* **539**, 541-545 (2016).

13 Khomyakov, P. A., Giovannetti, G., Rusu, P. C., Brocks, G., van den Brink, J. & Kelly, P. J. First-principles study of the interaction and charge transfer between graphene and metals. *Phys. Rev. B* **79**, 195425 (2009).

14 Hamada, I. & Otani, M. Comparative van der Waals density-functional study of graphene on metal surfaces. *Phys. Rev. B* **82**, 153412 (2010).

15 Gao, M., Pan, Y., Huang, L., Hu, H., Zhang, L. Z., Guo, H. M., Du, S. X. & Gao, H. J. Epitaxial growth and structural property of graphene on Pt(111). *Appl. Phys. Lett.* **98**, 033101 (2011).

16 Vanin, M., Mortensen, J. J., Kelkkanen, A. K., Garcia-Lastra, J. M., Thygesen, K. S. & Jacobsen, K. W. Graphene on metals: A van der Waals density functional study. *Phys. Rev. B* **81**, 081408 (2010).

17 Sutter, P., Sadowski, J. T. & Sutter, E. Graphene on Pt(111): Growth and substrate interaction. *Phys. Rev. B* **80**, 245411 (2009).

18 Morrow, B. H. & Striolo, A. Morphology and Diffusion Mechanism of Platinum Nanoparticles on Carbon Nanotube Bundles. *J. Phys. Chem. C* **111**, 17905-17913 (2007).

19 Sun, B., Ouyang, W., Gu, J., Wang, C., Wang, J. & Mi, L. Formation of Moiré superstructure of epitaxial graphene on Pt(111): A molecular dynamic simulation investigation. *Mater. Chem. Phys.* **253**, 123126 (2020).

20 Martínez, J. I., Merino, P., Pinardi, A. L., Gonzalo, O.-I., López, M. F., Méndez, J. & Martín-Gago, J. A. Role of the Pinning Points in epitaxial Graphene Moiré Superstructures on the Pt(111) Surface. *Sci. Rep.* **6**, 20354 (2016).

21 Giessibl, F. J. Advances in atomic force microscopy. *Rev. Mod. Phys.* **75**, 949-983 (2003).

22 Luan, B. & Robbins, M. O. The breakdown of continuum models for mechanical contacts. *Nature* **435**, 929-932 (2005).

23 Riedo, E., Gnecco, E., Bennewitz, R., Meyer, E. & Brune, H. Interaction Potential and Hopping Dynamics Governing Sliding Friction. *Phys. Rev. Lett.* **91**, 084502 (2003).

24 Gao, X., Ouyang, W., Urbakh, M. & Hod, O. Superlubric polycrystalline graphene interfaces. *Nat. Commun.* **12**, 5694 (2021).
