# Supplementary material for: Non-Amontons frictional behaviors of grain boundaries at layered material interfaces
Source: Nat Commun. 2024 Nov 2;15:9487. doi: 10.1038/s41467-024-53581-y (PMC11531579; doi:10.1038/s41467-024-53581-y)
Supplement: Supplementary file 3 — Description of Additional Supplementary Files [file 41467_2024_53581_MOESM3_ESM.pdf]

Description of Additional Supplementary Files

File Name: Supplementary Movie 1

Description: Sliding simulation of a $\theta_{\text{GB}} = 2°$ corrugated GB under zero normal load and zero temperature. To clearly demonstrate the buckling process, the PolyGr layer atoms are colored according to their out-of-plane position with respect to the average height of the two grains using the same color bar as in Fig. 4a of the main text. The bottom layer atoms in the diamond tip are depicted by partially transparent spheres to indicate the tip position. Under these conditions, the GB protrusion undergoes significant snap-through buckling when the tip approaches/leaves the grain boundary region.

File Name: Supplementary Movie 2

Description: Sliding simulation of a $\theta_{\text{GB}} = 2°$ corrugated GB under a normal load of 24.4 nN and zero temperature. The atoms are colored the same as in Supplementary Movie 1. Under these conditions, the GB protrusion does not experience abrupt snap-through buckling events upon tip sliding.

File Name: Supplementary Movie 3

Description: Sliding simulation of a $\theta_{\text{GB}} = 27.8°$ flat GB under zero normal load and zero temperature. The atoms are colored the same as in Supplementary Movie 1 but using the color bar appearing in Fig. 4b of the main text. Under these conditions, the GB protrusion does not experience abrupt snap-through buckling events upon tip sliding.
